# Compound Yeast Culture Reshapes Gut Microbiota and Functional Pathways to Enhance Antioxidant Capacity and Immune Homeostasis in Suckling Calves

**DOI:** 10.3390/microorganisms14050995

**Published:** 2026-04-29

**Authors:** Xueqiang Li, Xi Liang, Puguo Hao, Jingze Wu, Dacheng Liu

**Affiliations:** 1 College of Veterinary Medicine, Inner Mongolia Agricultural University, Hohhot 010018, China; lixueqiang2023@163.com (X.L.);; 2 National Center of Technology Innovation for Dairy, Hohhot 010018, China

**Keywords:** diarrhea, growth performance, immune response, metagenomics, oxidative stress, yeast-based additive

## Abstract

Diarrhea in suckling calves is associated with impaired growth, oxidative stress, immune dysfunction, and intestinal microbial dysbiosis. This study evaluated the effects of compound yeast culture (CYC) supplementation on growth performance, fecal characteristics, antioxidant capacity, immune function, and gut microbiota in diarrheic Holstein calves. Thirty-six approximately 7-day-old calves were enrolled, including 12 healthy calves (CON) and 24 diarrheic calves randomly assigned to a diarrhea group (DIA) or a CYC-supplemented group (DIA-YC; 50 g/d for 30 days). The experimental period lasted 60 days. Compared with the DIA group, calves in the DIA-YC group showed significantly higher average daily feed intake and average daily gain (ADG) during days 31–60 and across the entire period (*p* < 0.05), with a trend towards increased body weight. Fecal scores were significantly elevated in diarrheic calves during the early and mid-stages but were markedly reduced by CYC supplementation from days 7 to 30; no significant difference was observed between DIA-YC and CON during days 16–30 (*p* > 0.05). Diarrheic calves exhibited oxidative stress, characterized by decreased total antioxidant capacity (T-AOC) and increased malondialdehyde (MDA). CYC supplementation significantly increased T-AOC, superoxide dismutase (SOD), and glutathione peroxidase (GSH-Px) activities, while reducing MDA levels (*p* < 0.05). Immune analysis showed higher serum IgG and IL-10 levels and lower TNF-α levels in the DIA-YC group, along with improved intestinal barrier indicators, including diamine oxidase (DAO) activity and endotoxin levels. Metagenomic analysis revealed that diarrhea reduced microbial richness and diversity and altered community structure, whereas CYC partially restored microbial diversity and increased beneficial genera such as *Prevotella*, *Coprococcus*, *Ruminococcus*, and *Parabacteroides*. Functional analysis indicated that CYC enhanced pathways related to immune regulation, energy metabolism, and antioxidant function. CYC supplementation alleviates oxidative stress and immune dysfunction by modulating gut microbiota, thereby improving growth performance and reducing diarrheal severity in calves.

## 1. Introduction

The early life stage of calves represents a critical window for the rapid maturation of the digestive system, immune competence, and growth development; however, it is also a period of heightened susceptibility to gastrointestinal disorders, particularly diarrhea. Epidemiological evidence indicates that diarrhea is among the leading causes of early morbidity and mortality in calves, substantially increasing rearing costs and exerting long-term adverse effects on subsequent growth performance and production potential [1]. Following the onset of diarrhea, calves commonly experience fluid and electrolyte imbalances, reduced feed intake, and impaired nutrient absorption, which collectively disrupt body weight gain and metabolic homeostasis. Notably, even after the resolution of clinical symptoms, growth-restricting effects may persist [2]. Therefore, the development of safe and effective nutritional management strategies to mitigate calf diarrhea and its associated secondary damage is of considerable importance for improving calf health and production efficiency.

Accumulating evidence suggests that calf diarrhea is not merely a gastrointestinal manifestation but is accompanied by complex pathophysiological alterations, including disruption of intestinal barrier integrity, aberrant activation of inflammatory responses, and elevated oxidative stress [3]. During diarrheal episodes, pathogenic microorganisms and their metabolites compromise intestinal epithelial integrity, increase intestinal permeability, stimulate the release of pro-inflammatory mediators, and induce excessive production of reactive oxygen species, thereby establishing a vicious cycle characterized by ‘inflammation–oxidative stress–barrier dysfunction’ [4,5]. Concurrently, dysregulation of gut microbial composition and metabolic function has been recognized as a key intrinsic mechanism underlying the persistence of diarrhea and delayed recovery. Intestinal microbial imbalance not only weakens the colonization resistance and metabolic activities of beneficial microorganisms but also facilitates the expansion of potential pathogens, further aggravating inflammatory responses and systemic metabolic disturbances [6]. Consequently, nutritional strategies aimed at restoring intestinal microecological balance while enhancing antioxidant and immune defense capacities are considered pivotal for the prevention, control, and recovery from calf diarrhea [7].

Yeast cultures (YCs) are widely utilized as functional feed additives to improve feed intake, rumen fermentation efficiency, and growth performance in ruminants. Nevertheless, their efficacy in practical production systems remains inconsistent, largely due to variations in yeast strains, fermentation substrates, fermentation conditions, and processing techniques. These factors collectively contribute to substantial variability in the concentration and biological activity of functional components within YC, thereby influencing product stability and application outcomes [8]. The compound yeast culture (CYC) used in the present study is a probiotic formulation specifically developed by our research group for ruminants and was produced by the co-fermentation of two yeast strains, aiming to integrate the functional advantages of each strain. Through the targeted selection of yeast strains and optimization of fermentation substrates and process parameters, this formulation achieves the maximal accumulation of functional bioactive components, ensuring high active ingredient content while enhancing bioavailability and biological efficacy [9,10]. Previous studies conducted by our research group have demonstrated that this CYC significantly improves ADG and economic efficiency in meat sheep and beef cattle, while concurrently enhancing both specific and non-specific immune responses and reinforcing systemic antioxidant capacity [11,12]. Moreover, supplementation with this CYC in suckling Holstein calves markedly improved growth performance, strengthened antioxidant defense systems, enhanced immune function, and optimized the structure of the intestinal microbial community [13]. Collectively, these findings provide a robust theoretical and experimental foundation for the application of this compound yeast culture in diarrheic calves.

However, the systematic mechanisms through which compound yeast culture exerts its effects under the specific physiological conditions associated with calf diarrhea remain insufficiently elucidated, particularly with respect to the integrated regulation of growth performance, antioxidant status, immune function, and gut microbial structure and function. Accordingly, the present study aimed to systematically evaluate the effects of CYC supplementation on growth performance, antioxidant capacity, immune function, and the composition and functional potential of the gut microbiota in suckling Holstein calves. This study further sought to elucidate the potential mechanisms by which CYC alleviates diarrhea and promotes health recovery through coordinated modulation of the ‘gut microbiota–functional pathways–host phenotype’ axis, thereby providing scientific evidence to support the application of CYC in sustainable and health-oriented ruminant production systems.

## 2. Materials and Methods

### 2.1. Preparation of Compound Yeast Culture

Proprietary strains of *Saccharomyces cerevisiae* XR4 and *Kluyveromyces marxianus* BC were selected by the research group and blended at a 1:1 ratio to establish a fermentation starter culture, with a viable cell concentration of 1.5 × 10^8^ cfu/g. The mixed culture was inoculated at 10% (*w*/*w*) of the solid substrate, and sterile water was added to adjust the initial moisture content of the fermentation substrate to 38–40%. The formulation of the fermentation substrate is provided in Table A1 [9]. Solid-state pile fermentation was performed in a dedicated fermentation workshop, with the pile height maintained at 60–65 cm. Substrate temperature was monitored at 3 h intervals throughout the fermentation process. After 24 h of fermentation, once the substrate temperature exceeded 40 °C, the pile was turned once to ensure uniform fermentation. Fermentation was then continued for a total duration of 72 h. Upon completion, the fermented material was subjected to low-temperature drying, followed by grinding and packaging to obtain the final compound yeast culture product. The nutritional composition of the compound yeast culture medium is presented in Table A2 [10,14].

### 2.2. Animals, Diets, and Experimental Design

#### 2.2.1. Experimental Design and Animals

The experiment was conducted at Ainiu Animal Husbandry Co., Ltd. (Suihua, Heilongjiang, China), a commercial dairy farm located in Suihua, Heilongjiang Province, China. All calves used in this study were sourced from this farm. The experimental protocol was approved by the Animal Welfare and Ethics Committee of Inner Mongolia Agricultural University (approval no. NND2023123), and all procedures were carried out in accordance with the guidelines for the care and use of laboratory animals issued by the National Research Council (2022-6-10/SYXK 2022-0031).

A total of 36 Holstein calves (approximately 7 d of age, from the same batch and with similar initial body weight) were enrolled in this study. At the beginning of the experiment (Day 0), twelve calves with normal fecal consistency and no clinical signs of diarrhea were assigned to the healthy control group (CON). The remaining 24 calves were naturally diarrheic and were enrolled in the diarrhea-related experimental treatments.

The total experimental period lasted 60 d. Throughout the entire study, growth performance and fecal scores were continuously recorded for all calves. It should be noted that some calves initially assigned to the CON group developed diarrhea during the experimental period; for these calves, growth performance and fecal score assessments were continued. However, for subsequent analyses of serum antioxidant capacity, immune function indicators, and fecal microbiota, only calves that remained free of diarrhea throughout the experimental period were selected for sample collection and analysis.

#### 2.2.2. Case Definition of Diarrhea

Diarrhea was assessed using a standardized fecal scoring system based on a 5-point scale, where 1 = normal, well-formed feces and 5 = watery feces. A calf was diagnosed as diarrheic when a fecal score ≥ 3 was observed on two consecutive occasions within a 24 h period. The day on which diarrhea was first diagnosed was defined as Day 0. All evaluations were performed by a licensed veterinarian to ensure consistency and accuracy. While this clinical scoring system is widely accepted in calf studies, it should be noted that no laboratory-based quantitative pathogen detection (e.g., enumeration of fecal bacteria) was performed in this study.

#### 2.2.3. Randomization and Allocation

On the day of diarrhea diagnosis (Day 0), diarrheic calves were stratified according to age at the onset of diarrhea and fecal score severity. Subsequently, calves were randomly allocated to either the diarrheic control group (DIA) or the yeast culture–supplemented group (DIA-YC), with 12 calves per group, using a computer-generated random number table. Randomization was performed by an investigator who was not involved in daily animal management or outcome measurements to minimize allocation bias.

#### 2.2.4. Blinding and Outcome Assessment

Due to the nature of dietary supplementation, blinding of animal caretakers was not feasible. However, fecal scoring was independently conducted by licensed veterinarians at the farm according to standardized scoring criteria to reduce observer bias. Laboratory analyses of blood and fecal samples were performed with investigators blinded to treatment allocation during the analytical phase.

#### 2.2.5. Dietary Treatments and Management

All calves were housed individually in pens measuring 2.2 × 2.0 m under identical environmental and sanitary conditions. Calves were fed whole milk twice daily at 07:30 and 16:30, with feeding volumes adjusted according to age (Table 1). Calf starter feed was offered ad libitum beginning at 7 d of age, and fresh water was freely available throughout the experiment.

The starter feed was formulated to meet or exceed the nutrient requirements of dairy calves as recommended by the NRC (Nutrient Requirements of Dairy Cattle, 2001) [15]. The ingredient composition and nutrient levels of the starter feed are presented in Table 2. Dry matter (DM) content was determined by oven drying at 105 °C to a constant weight, following the AOAC (2005) Method 934.01 [16]. Crude protein (CP) content was analyzed using the Kjeldahl method according to AOAC (2005) Method 990.03 [16]. Neutral detergent fiber (NDF) and acid detergent fiber (ADF) contents were determined using the procedures described by Van Soest et al. (1991) [17].

During the experimental period, calves in the CON and DIA groups received 50 g/d of unfermented culture substrate, whereas calves in the DIA-YC group received 50 g/d of yeast culture. Dietary supplementation was administered for 30 d, whereas growth performance and health status were monitored throughout the 60 d experimental period.

#### 2.2.6. Supportive Therapy and Health Management

All diarrheic calves received standard supportive care according to routine farm veterinary practices, including oral electrolyte supplementation when clinically indicated. No additional antibiotics or pharmacological treatments were administered during the experimental period. If individual calves developed severe systemic symptoms requiring intensive medical intervention, their growth performance and fecal scores continued to be recorded; however, blood and fecal samples from these calves were excluded from analyses of antioxidant capacity, immune function, and intestinal microbiota. Accordingly, blood and fecal samples for these analyses were collected only from six calves per group that did not receive antibiotics or intensive medical interventions during the experimental period.

#### 2.2.7. Experimental Timeline and Sampling

The total experimental duration was 60 d. For diarrheic calves, Day 0 was defined as the day of diarrhea diagnosis, and animals were monitored continuously until Day 60. Healthy control calves were assessed for growth performance and fecal scores at corresponding chronological ages. Blood samples for antioxidant and immune parameter analyses and fecal samples for microbiota analysis were collected only from calves meeting the predefined experimental criteria, as described above.

### 2.3. Sample Collection

#### 2.3.1. Collection of Serum Samples

On days 1, 7, 15, and 30 of the experimental period, approximately 10 mL of blood was collected from the jugular vein of each calf into procoagulant-containing vacuum tubes prior to the morning feeding. Blood samples were centrifuged at 3000 rpm for 10 min to obtain serum. The serum was subsequently aliquoted into 2 mL sterile, enzyme-free cryogenic vials, properly labelled, and stored at −80 °C until further analysis. Serum samples were used to determine the concentrations of immunoglobulin G (IgG), immunoglobulin A (IgA), interleukin-2 (IL-2), interleukin-4 (IL-4), interleukin-10 (IL-10), tumor necrosis factor-α (TNF-α), diamine oxidase (DAO), endotoxin (ET), total antioxidant capacity (T-AOC), superoxide dismutase (SOD), glutathione peroxidase (GSH-Px), and malondialdehyde (MDA).

#### 2.3.2. Collection of Fecal Samples

Fresh fecal samples were collected from six calves randomly selected from each group at the beginning of the trial (day 0) and on day 30 of the experimental period. Sample collection was performed using sterile disposable gloves, and approximately 10 g of fecal material was obtained directly from the rectum of each calf to minimize environmental contamination. The samples were immediately transferred into sterile centrifuge tubes and snap-frozen in liquid nitrogen. Subsequently, all samples were stored at −80 °C until DNA extraction and metagenomic sequencing analyses.

### 2.4. Index Measurement and Methods

#### 2.4.1. Measurement of Growth Performance Indexes

Calves were weighed on days 1, 30, and 60 of the experimental period after an overnight fast and prior to the morning feeding. Daily starter feed was offered and refusals were recorded to determine individual starter feed intake. ADG and average daily starter feed intake were subsequently calculated for each calf.

The relevant calculation formulas are as follows:

ADFI (g/d) = [total feed amounts (g) − total residue amounts (g)]/trial days

ADG (kg/d) = (final body weights − initial body weights)/trial days

#### 2.4.2. Fecal Scoring

Throughout the experimental period, the fecal consistency of each calf was assessed twice daily at 07:00 and 18:00 h. Fecal scores were assigned according to the method described by Liang et al., with detailed scoring criteria provided in Table 3. A fecal score of ≥3 was defined as diarrhea.

#### 2.4.3. Determination of Antioxidant Capacity Indexes

The methods and kit information used for the determination of serum T-AOC, SOD, GSH-Px, and MDA levels in calves are provided in Table 4. All assay kits were obtained from Ruixin Biological, and the analytical procedures were performed in strict accordance with the manufacturer’s instructions.

#### 2.4.4. Determination of Immune Function Indexes

The methods and kit information for the measurement of serum IgG, IgA, IL-2, IL-4, IL-10, TNF-α, ET, and DAO are summarized in Table 5. All kits were purchased from Ruixin Biological, and all assays were conducted following the manufacturer’s protocols.

#### 2.4.5. Metagenomic Sequencing and Bioinformatic Analysis

Total genomic DNA was extracted from fecal samples using the E.Z.N.A.^®^ Soil DNA Kit (Omega Bio-tek, Norcross, GA, USA), according to the manufacturer’s protocol. DNA integrity was evaluated by 1% agarose gel electrophoresis, and DNA concentration and purity were assessed spectrophotometrically. Purified DNA was subsequently fragmented to an average size of approximately 350 bp using a Covaris M220 ultrasonicator, and paired-end sequencing libraries were constructed using the NEXTFLEX Rapid DNA-Seq Kit (Bioo Scientific, Austin, TX, USA).

Metagenomic sequencing was performed on the Illumina NovaSeq™ X Plus platform (Illumina, San Diego, CA, USA), with sequencing services provided by Shanghai Majorbio Bio-Pharm Technology Co., Ltd. (Shanghai, China) Raw sequencing reads were initially aligned to the host genome using a Burrows–Wheeler Aligner (BWA, v0.7.17) [19] to remove host-derived sequences. High-quality, non-host reads were then assembled de novo using MEGAHIT software (v1.1.2) [20], and contigs with a minimum length of 300 bp were retained for downstream analyses.

Open reading frames (ORFs) were predicted from the assembled contigs using Prodigal software (v2.6.3), and gene sequences with lengths ≥ 100 bp were selected for further analysis. The predicted nucleotide sequences were translated into their corresponding amino acid sequences. All predicted amino acid sequences from each sample were clustered using CD-HIT software (v4.7) [21], with a sequence identity threshold of 90% and a coverage threshold of 90%. The longest sequence from each cluster was selected to generate a non-redundant gene catalogue.

High-quality reads from each sample were mapped back to the non-redundant gene catalogue using SOAPaligner software (v2.21) [22], with a sequence identity threshold of 95% to quantify gene abundance. For taxonomic annotation, amino acid sequences from the non-redundant gene set were aligned against the NCBI non-redundant (NR) protein database using DIAMOND software (v2.0.13) [23] in BLASTP mode, with an e-value cutoff of 1 × 10^−5^. Taxonomic assignments were determined based on the NR annotation results, and the relative abundance of each microbial species was calculated as the sum of the abundances of its corresponding genes.

### 2.5. Statistical Analysis

All data were compiled using Microsoft Excel 2010 and analyzed with IBM SPSS Statistics 22.0 (IBM Corp., Armonk, NY, USA). Growth performance and fecal scores measured during the 60 d experimental period were analyzed using a mixed-effects model, with treatment as a fixed effect and calf as a random effect.

Serum antioxidant capacity, immune function parameters, and fecal microbiota-related variables measured at single time points were analyzed by one-way analysis of variance (ANOVA). When significant effects were detected, mean differences were compared using Tukey’s post hoc test.

Data are presented as means ± standard error of the mean (SEM). Spearman correlation analysis was performed to evaluate relationships between differential KEGG functional pathways and serum antioxidant and immune indicators based on pathway annotations from KEGG [24]. Statistical significance was declared when *p* < 0.05.

## 3. Results

### 3.1. Effects of Compound Yeast Culture on Growth Performance of Calves

As shown in Table 6, no significant differences in average daily feed intake (ADFI) were observed among the groups during days 1–30, although the DIA group consistently exhibited the lowest intake. During days 31–60, calves in the DIA-YC group showed a significantly higher ADFI than those in the DIA group (*p* < 0.05). Over the entire experimental period (days 1–60), ADFI in the DIA-YC group exceeded that of the DIA and CON groups by 29.40 g/d and 19.37 g/d, respectively, corresponding to increases of 15.28% and 9.57% (*p* < 0.05), which may be attributed to improved gut health and reduced diarrhea, leading to enhanced feed intake. No significant differences in body weight were detected among groups at any sampling point; however, at day 60, calves in the DIA-YC group tended to exhibit slightly higher body weights than those in the DIA and CON groups. Similarly, ADG did not differ significantly among groups during days 1–30, with the lowest values observed in the DIA group. From days 31–60, ADG in the DIA-YC group was significantly increased by 10.47% compared with the DIA group (*p* < 0.05). Across the entire experimental period, calves in the DIA-YC group achieved an ADG of 0.83 kg/d, which was significantly higher than that of the DIA group (*p* < 0.05), representing an increase of 6.41%. This improvement may be attributed to enhanced gut health and nutrient utilization, as yeast culture stabilizes intestinal microbiota, improves digestion and absorption, and reduces inflammation-related energy expenditure.

Collectively, these results demonstrate that compound yeast culture supplementation effectively enhances feed intake and contributes to improved growth performance in diarrheic calves.

### 3.2. Effect of Compound Yeast Culture on Faecal Scores in Calves

To assess the effects of compound yeast culture supplementation on fecal characteristics in diarrheic calves, fecal scores were analyzed across different experimental periods, and the results are presented in Table 7. During days 1–7, fecal scores in both the DIA and DIA-YC groups were significantly higher than those in the CON group (*p* < 0.01), whereas no significant difference was observed between the DIA and DIA-YC groups. From days 7 to 15, fecal scores differed significantly among all three groups (*p* < 0.01), with the lowest values observed in the CON group, the highest in the DIA group, and intermediate values in the DIA-YC group. Pairwise comparisons indicated significant differences between each group (*p* < 0.05). During days 16–30, the DIA group exhibited significantly higher fecal scores than both the CON and DIA-YC groups (*p* < 0.05), while no significant difference was detected between the DIA-YC and CON groups. From days 31 to 60, fecal scores did not differ significantly among the three groups.

Overall, calves in the DIA group consistently displayed elevated fecal scores compared with the healthy CON group during the early and mid-experimental phases. In contrast, compound yeast culture supplementation was associated with reduced fecal scores in the DIA-YC group during the 7–15 and 16–30 day periods. Notably, during days 16–30, fecal scores in the DIA-YC group were comparable to those of the CON group. These temporal changes indicate that compound yeast culture supplementation contributed to a gradual alleviation of diarrhea severity in affected calves. By the later stages of the trial, fecal scores converged among groups, suggesting a normalization of intestinal function.

Collectively, these findings indicate that compound yeast culture supplementation supports intestinal health and reduces the incidence and severity of diarrhea in calves.

### 3.3. Effects of Compound Yeast Culture on Antioxidant Capacity in Calves

To evaluate the effects of compound yeast culture supplementation on oxidative stress status in diarrheic calves, serum T-AOC, SOD, GSH-Px, and MDA concentrations were determined at multiple time points throughout the experimental period. The results are summarized in Table 8.

On day 1, no significant differences were detected among groups in serum T-AOC, SOD, or MDA levels. In contrast, GSH-Px activity in both the DIA and DIA-YC groups was significantly higher than that in the CON group (*p* < 0.05). By day 7, T-AOC was significantly reduced in the DIA group compared with the CON group (*p* < 0.05), whereas the DIA-YC group exhibited intermediate values. Serum SOD activity was markedly elevated in both the DIA and DIA-YC groups relative to the CON group (*p* < 0.001). Additionally, GSH-Px activity in the DIA-YC group was significantly higher than that in the CON group (*p* < 0.05), while MDA concentrations in both the DIA and DIA-YC groups exceeded those observed in the CON group (*p* < 0.001).

On day 15, serum T-AOC in the DIA group remained significantly lower than that in both the CON and DIA-YC groups (*p* < 0.01), with no significant difference observed between the DIA-YC and CON groups. The activities of SOD and GSH-Px were significantly higher in both the DIA and DIA-YC groups compared with the CON group (*p* < 0.01). Moreover, MDA levels were significantly elevated in the DIA group relative to the CON group (*p* < 0.01), whereas the DIA-YC group exhibited intermediate values. By day 30, T-AOC in the DIA-YC group was significantly higher than that in both the CON and DIA groups (*p* < 0.01). SOD and GSH-Px activities in the DIA and DIA-YC groups remained significantly greater than those in the CON group (*p* < 0.001). In contrast, MDA concentrations in the DIA group were significantly higher than those in the CON group (*p* < 0.05), while no significant difference was detected between the DIA-YC and CON groups.

Collectively, diarrheic calves showed increased oxidative stress during the early stages of the trial, while compound yeast culture supplementation was associated with higher antioxidant enzyme activities, improved systemic antioxidant indicators, and reduced lipid peroxidation levels.

### 3.4. Effects of Compound Yeast Culture on Immune Function in Calves

To assess the regulatory effects of compound yeast culture supplementation on humoral immunity, inflammatory responses, and intestinal barrier function in calves with diarrhea, serum immunoglobulins, cytokines, and intestinal permeability-related indicators were comparatively analyzed among the experimental groups at multiple time points. The results are presented in Table 9.

At day 0, serum IgG concentrations were significantly higher in the CON group than in both the DIA and DIA-YC groups (*p* < 0.05). No significant differences in IgG levels were observed among groups at days 7 and 15. By day 30, IgG concentrations in the DIA-YC group were significantly higher than those in the DIA group (*p* < 0.05), with the CON group exhibiting intermediate values. Serum IgA levels did not differ significantly among groups at days 0, 7, or 15. However, at day 30, IgA levels in the DIA group were significantly higher than those in both the CON and DIA-YC groups (*p* < 0.05).

With respect to cytokine profiles, no significant differences in IL-2 concentrations were detected among groups at days 0 and 7. At day 15, the DIA-YC group exhibited significantly higher IL-2 levels than the DIA group (*p* < 0.05). By day 30, IL-2 concentrations in the DIA group were significantly lower than those in both the CON and DIA-YC groups (*p* < 0.05), whereas no significant difference was observed between the CON and DIA-YC groups. Serum TNF-α levels at days 0 and 7 were significantly elevated in both the DIA and DIA-YC groups compared with the CON group (*p* < 0.05). At day 30, TNF-α levels in the DIA group remained significantly higher than those in both the CON and DIA-YC groups (*p* < 0.05). Serum IL-10 concentrations did not differ significantly among groups at day 0. At days 7 and 15, IL-10 levels in both the DIA and DIA-YC groups were significantly higher than those in the CON group (*p* < 0.05). At day 30, the DIA-YC group exhibited significantly higher IL-10 concentrations than both the CON and DIA groups (*p* < 0.05). In contrast, IL-4 levels showed no significant differences among groups at any time point during the trial.

Regarding intestinal barrier function, serum DAO and ET concentrations at day 0 were significantly higher in both the DIA and DIA-YC groups compared with the CON group (*p* < 0.05). At day 7, DAO and ET levels in the DIA group remained significantly elevated relative to the CON group (*p* < 0.05), whereas no significant differences were detected between the DIA-YC group and the other two groups. At days 15 and 30, no significant intergroup differences were observed for DAO or ET.

Overall, diarrheic calves exhibited altered immune responses, increased inflammatory indicators, and impaired intestinal barrier function. Compound yeast culture supplementation increased humoral immune parameters, modulated cytokine profiles, and improved intestinal barrier-related indicators.

### 3.5. Effects of Compound Yeast Culture on the Structural Composition of the Calf Gut Microbiome

**Figure 1:**
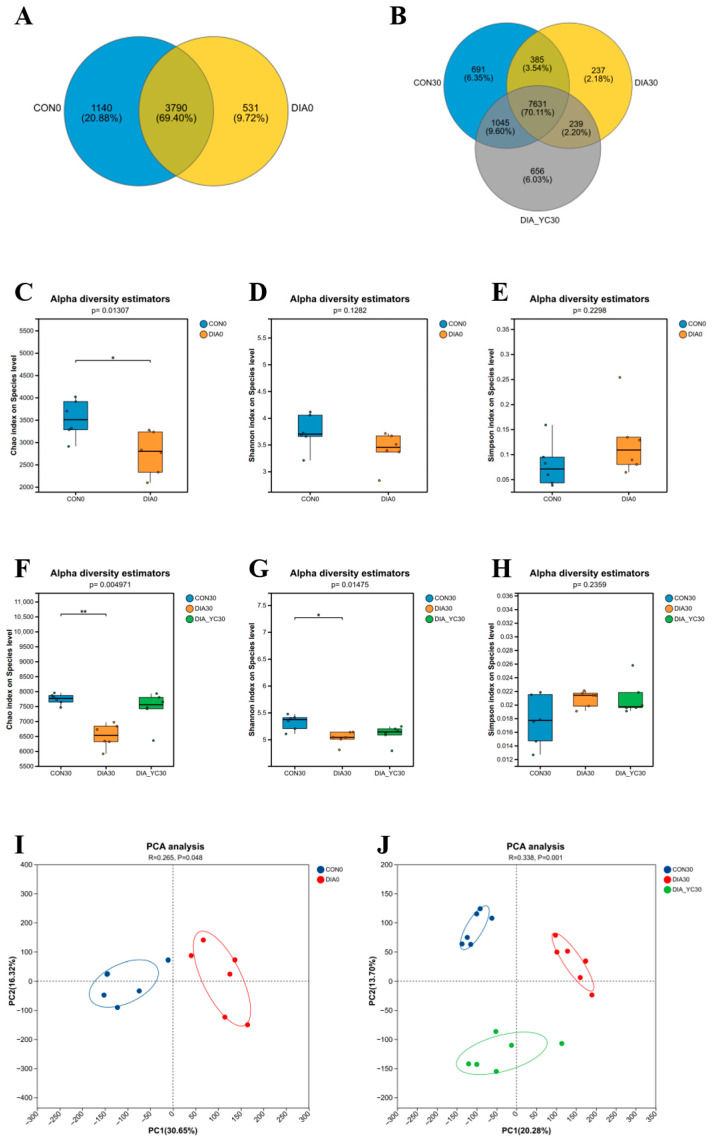
Effects of diarrhea and compound yeast culture supplementation on intestinal microbiota diversity and community structure in calves. Venn diagrams illustrate the shared and unique amplicon sequence variants (ASVs) among groups on d 0 ((**A**) CON vs. DIA) and d 30 ((**B**) CON, DIA, and DIA-YC). Alpha diversity indices, including Chao1 richness (**C**,**F**), Shannon diversity (**D**,**G**), and Simpson index (**E**,**H**), were used to evaluate fecal microbial richness and diversity on d 0 (**C**–**E**) and d 30 (**F**–**H**). Beta diversity was assessed by principal component analysis (PCA) based on Euclidean distance, showing differences in overall fecal microbial community structure between CON and DIA on d 0 (**I**) and among CON, DIA, and DIA-YC on d 30 (**J**). Statistical differences in community structure were evaluated using ANOSIM. *n* = 6. *p* < 0.05 (*), *p* < 0.01 (**).

To explore the effects of compound yeast culture supplementation on the structural composition of the calf gut microbiome, fecal samples were subjected to metagenomic sequencing using the Illumina NovaSeq™ X Plus platform (Illumina, USA). Venn diagram analysis revealed that on day 0 of the trial, a total of 5461 amplicon sequence variants (ASVs) were identified in samples from the healthy control calves (CON group) and diarrheic calves (DIA group) (Figure 1A). Among these, 3790 ASVs were shared between the two groups, accounting for 69.40% of the total ASVs. In addition, the CON and DIA groups harbored 1140 (20.88%) and 531 (9.72%) unique ASVs, respectively. By day 30 of the trial, a total of 10,884 ASVs were detected across samples from the CON, DIA, and compound yeast culture-supplemented diarrheic calves (DIA-YC group) (Figure 1B). Of these, 7631 ASVs were common to all three groups, representing 70.11% of the total ASVs. Meanwhile, 691 (6.35%), 237 (2.18%), and 656 (6.03%) ASVs were uniquely identified in the CON, DIA, and DIA-YC groups, respectively.

α-diversity analysis demonstrated that Good’s coverage exceeded 99.5% for all samples, indicating that sequencing depth was sufficient to accurately characterize the microbial communities present in the fecal samples. At day 0, the Chao richness index of the fecal microbiota in diarrheic calves was significantly lower than that of the healthy control calves (*p* < 0.05) (Figure 1C). In parallel, the Shannon diversity index exhibited a decreasing trend (Figure 1D), while the Simpson index showed an increasing trend (Figure 1E), although these differences were not statistically significant. These results suggest that diarrhea primarily reduced microbial richness during the early stage of the trial, while overall community diversity remained relatively stable. By day 30, diarrheic calves in the DIA group exhibited significantly reduced microbial richness (Chao index; Figure 1F) and diversity (Shannon index; Figure 1G) compared with the healthy CON group (*p* < 0.05). In contrast, calves in the DIA-YC group showed higher richness and diversity indices than those in the DIA group, although the differences did not reach statistical significance. Concurrently, the Simpson index in the DIA group was higher than that in the CON group (Figure 1H), albeit without a significant difference. These findings indicate that prolonged diarrhea markedly suppressed both richness and diversity of the gut microbiota, whereas compound yeast culture supplementation partially alleviated these detrimental effects.

Based on the observed alterations in α-diversity, β-diversity analysis was further performed to assess changes in overall microbial community structure among the treatment groups. Principal component analysis (PCA) based on Euclidean distance revealed a clear separation between the CON and DIA groups at day 0 (Figure 1I). This separation was supported by ANOSIM analysis, which confirmed a significant difference in gut microbial community structure between the two groups (*p* < 0.05). By day 30, distinct clustering patterns were observed among the CON, DIA, and DIA-YC groups in the PCA plot (Figure 1J), indicating pronounced differences in microbial community structure. Consistently, ANOSIM analysis demonstrated significant structural divergence among the three groups (*p* < 0.05).

**Figure 2:**
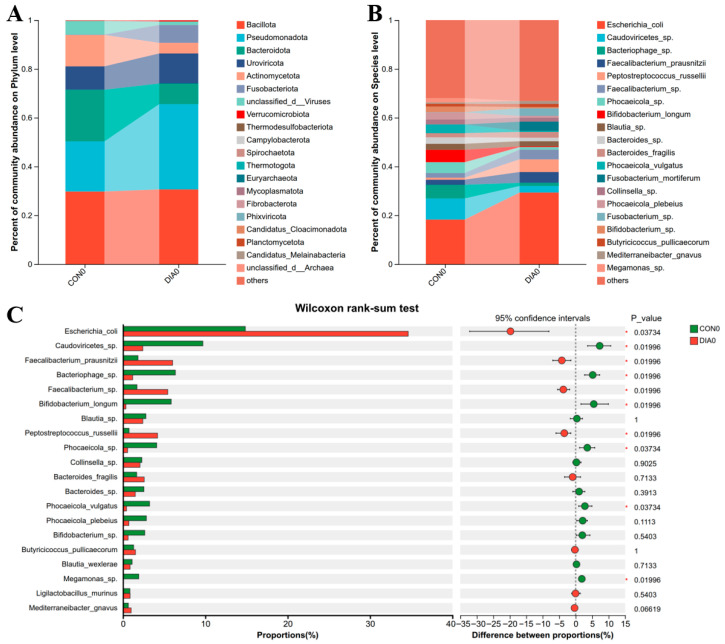
Effects of diarrhea on intestinal microbial composition in calves on d 0. Relative abundances of dominant bacterial phyla (**A**) and dominant species (**B**) in fecal samples from healthy control calves (CON) and diarrheic calves (DIA) on d 0. Differentially abundant species between CON and DIA were identified using the Wilcoxon rank-sum test, and the top 20 species at the species level are shown (**C**). Statistical significance was defined as *p* < 0.05 (*), and each group contained six biological replicates (*n* = 6).

On trial day 0, the gut microbiota of both healthy control calves (CON group) and diarrheic calves (DIA group) was dominated at the phylum level by Bacillota, Pseudomonadota, and Bacteroidota (Figure 2A). At the species level, the predominant taxa were *Escherichia coli*, unclassified Caudoviricetes, and unclassified Bacteriophages (Figure 2B).

Wilcoxon rank-sum analysis of the top 20 taxa at the species level revealed pronounced compositional differences between the two groups. Specifically, the relative abundances of *E. coli*, *Faecalibacterium prausnitzii*, *Faecalibacterium* sp., and *Peptostreptococcus russellii* were significantly elevated in the DIA group compared with the CON group. In contrast, diarrheic calves exhibited significantly reduced relative abundances of unclassified Caudoviricetes, unclassified Bacteriophages, *Bifidobacterium longum*, *Phocaeicola* sp., *Phocaeicola vulgatus*, and *Megamonas* sp. (Figure 2C).

**Figure 3:**
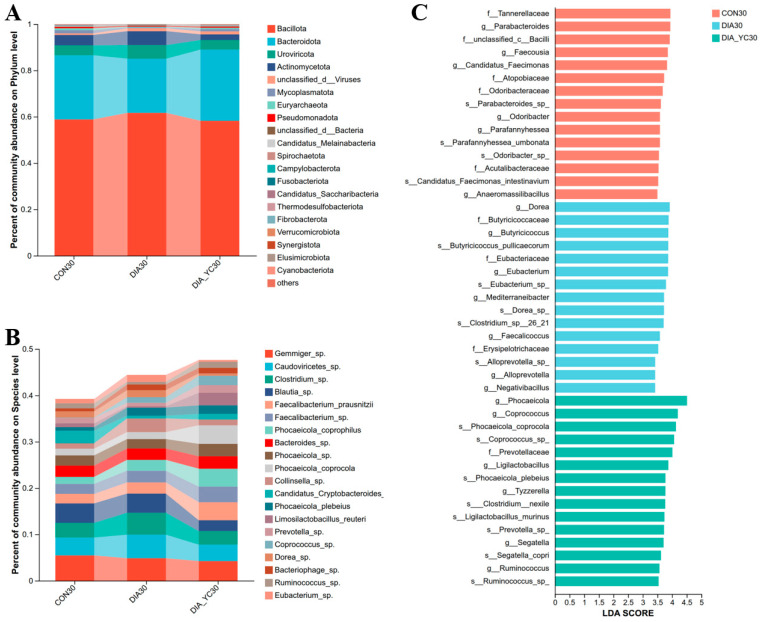
Effects of diarrhea and compound yeast culture supplementation on intestinal microbial composition in calves on d 30. Relative abundances of dominant bacterial and viral phyla (**A**) and dominant species (**B**) in fecal samples from healthy control calves (CON), diarrheic calves (DIA), and diarrheic calves supplemented with compound yeast culture (DIA-YC) on d 30. Differentially abundant species among groups were identified using linear discriminant analysis effect size (LEfSe) with LDA score ≥ 3 and *p* < 0.05, showing the top 30 species at the species level (**C**). *n* = 6.

On day 30 of the trial, Bacillota, Bacteroidota, and Uroviricota were the dominant phyla in samples from the healthy calf control group (CON group), the diarrheic calf group (DIA group), and the diarrheic calf group supplemented with compound yeast culture (DIA-YC group) (Figure 3A). The dominant species were *Gemmiger* sp., unclassified Caudoviricetes, and *Clostridium* sp. (Figure 3B).

LEfSe differential discriminant analysis of the top 30 species revealed that, when the LDA effect size was ≥3 and *p* < 0.05, compared with the CON group, the relative abundances of *Phocaeicola plebeius*, *Butyricicoccus pullicaecorum*, and *[Clostridium] nexile* were significantly increased, while *Ruminococcus* sp. and *Parabacteroides* sp. were significantly decreased (Figure 3C). Compared with the DIA group, the relative abundances of *Phocaeicola coprocola*, *Prevotella* sp., *Coprococcus* sp., *Ruminococcus* sp., and *Parabacteroides* sp. were significantly increased in the DIA-YC group, whereas *Dorea* sp., *Eubacterium* sp., and *B. pullicaecorum* were significantly decreased (Figure 3C).

### 3.6. Correlation Between Gut Microbiota and Serum Antioxidant and Immune Parameters

**Figure 4:**
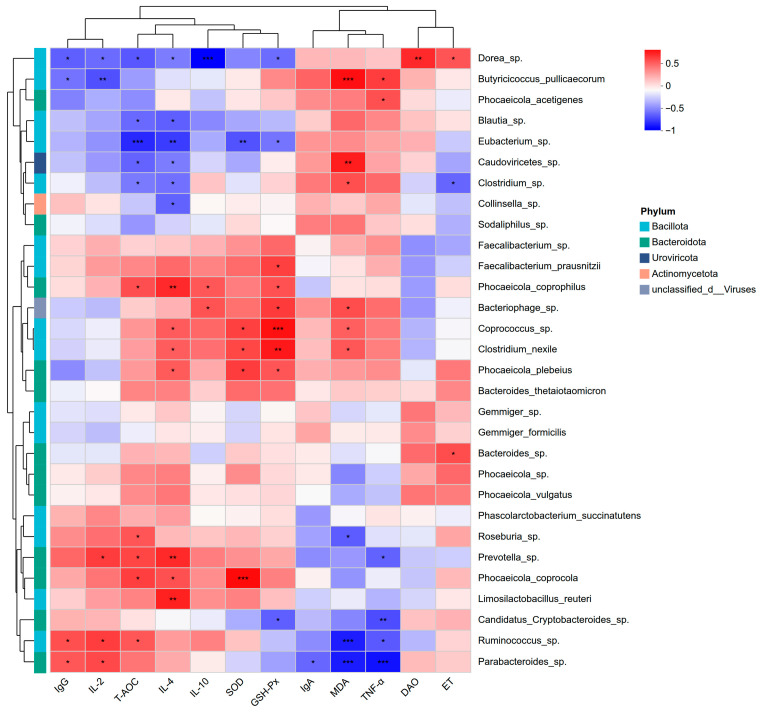
Spearman correlation between intestinal dominant species and serum antioxidant and immune parameters in calves. Spearman correlation analysis was performed to evaluate associations between dominant fecal bacterial species and serum antioxidant (T-AOC, SOD, GSH-Px, MDA) and immune parameters (IgG, IgA, IL-2, IL-4, IL-10, TNF-α, DAO, ET). Correlations are visualized as a heatmap, with positive correlations shown in red and negative correlations in blue. Statistical significance was indicated as *p* < 0.05 (*), *p* < 0.01 (**), and *p* < 0.001 (***); *n* = 6 per group.

To further elucidate the associations between gut microbiota and host antioxidant capacity as well as immune function, a Spearman correlation heatmap was constructed to evaluate the relationships between serum antioxidant and immune parameters and the dominant bacterial taxa detected in fecal samples (Figure 4).

The results showed that *Phocaeicola_coprocola* was positively correlated with serum T-AOC and SOD, as well as the anti-inflammatory cytokine IL-4. *Prevotella*_sp. exhibited positive correlations with T-AOC, IL-2 and IL-4, while showing a negative correlation with the pro-inflammatory cytokine TNF-α. *Coprococcus*_sp. was positively associated with SOD, GSH-Px, and IL-4. In addition, *Ruminococcus*_sp. showed positive correlations with T-AOC, IgG, and IL-2, and negative correlations with MDA and TNF-α. Similarly, *Parabacteroides*_sp. was positively correlated with IgG and IL-2, while negatively correlated with MDA and TNF-α.

Conversely, *Dorea*_sp. exhibited negative correlations with T-AOC, GSH-Px, IgG, IL-2, IL-4, and IL-10, but was positively correlated with serum DAO and ET. *Eubacterium*_sp. showed negative correlations with T-AOC, SOD, GSH-Px, and IL-4. Moreover, *Butyricicoccus_pullicaecorum* was positively correlated with MDA and TNF-α, and negatively correlated with IgG and IL-2.

### 3.7. KEGG Functional Enrichment Analysis

To explore the functional implications of compound yeast culture on calf gut microbiota, KEGG functional enrichment analysis was performed across treatment groups. At day 30, the gut microbiota of diarrheic calves (DIA) exhibited marked functional alterations compared with healthy controls (CON), predominantly in pathways associated with immune defense and antioxidant mechanisms. Specifically, the Pathogenic *E. coli* infection pathway, related to pathogen invasion, was significantly enriched. Additional changes were observed in pathways involved in bacterial motility and ET synthesis, including Flagellar assembly and Lipopolysaccharide biosynthesis. Antioxidant-related functions, such as the Peroxisome pathway, were significantly downregulated, whereas pathways linked to reactive oxygen species metabolism and oxidative stress regulation—e.g., Chemical carcinogenesis–reactive oxygen species and Selenocompound metabolism—were substantially enriched. These findings indicate that diarrhea disrupts the functional homeostasis and redox balance of the calf gut microbiota (File S1).

To evaluate the restorative effects of compound yeast culture on microbial function, the DIA-YC group was compared with the DIA group. KEGG analysis revealed significant enrichment of multiple immune-regulatory and metabolic pathways in the DIA-YC group. Immune-related pathways, including the T cell receptor signaling pathway, the phagosome, and the TGF-β signaling pathway, were notably enhanced, suggesting improved gut immune regulatory capacity. In parallel, pathways associated with energy metabolism and antioxidant function—such as Oxidative phosphorylation, Riboflavin metabolism, and Ubiquinone and other terpenoid-quinone biosynthesis—were significantly upregulated. These results indicate that compound yeast culture supplementation promotes the recovery of gut microbial function, steering the microbiota of diarrheic calves towards a healthier state (File S2).

### 3.8. Correlation Analysis Between KEGG Functional Pathways and Host Antioxidant and Immune Indicators

**Figure 5:**
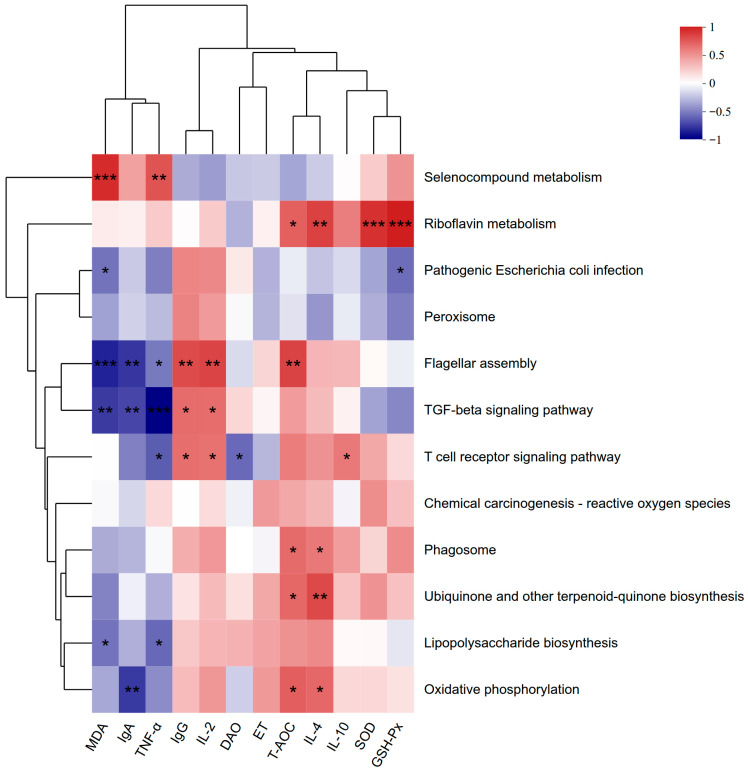
Spearman correlations between differential KEGG functional pathways and serum antioxidant and immune parameters in calves. Spearman correlation analysis was performed to assess the relationships between differentially enriched KEGG functional pathways and serum antioxidant (T-AOC, SOD, GSH-Px, MDA) and immune-related parameters (IgA, IgG, IL-2, IL-4, IL-10, TNF-α, DAO, ET). Correlations are presented as a heatmap, with red and blue indicating positive and negative correlations, respectively. Statistical significance was indicated as *p* < 0.05 (*), *p* < 0.01 (**), and *p* < 0.001 (***); *n* = 6 per group.

To further elucidate the associations between gut microbial functional alterations and host antioxidant capacity and immune function, Spearman correlation analysis was performed to assess relationships between serum antioxidant and immune parameters and differentially expressed KEGG functional pathways (Figure 5).

Among pathogen-related functional pathways, the Pathogenic *E. coli* infection pathway showed negative correlations with MDA and GSH-Px levels. The Flagellar assembly pathway was negatively correlated with MDA, TNF-α, and IgA, while positively correlated with IgG, IL-2, and T-AOC. In addition, the Lipopolysaccharide biosynthesis pathway exhibited negative correlations with MDA and TNF-α.

Regarding antioxidant- and metabolism-related pathways, no significant correlations were observed between the Peroxisome and Chemical carcinogenesis–reactive oxygen species pathways and the measured serum indicators. The Selenocompound metabolism pathway was negatively correlated with MDA and TNF-α. The Oxidative phosphorylation pathway showed a negative correlation with IgA but a positive correlation with IL-4 and T-AOC. Furthermore, the Riboflavin metabolism pathway was positively correlated with IL-4, T-AOC, SOD, and GSH-Px, while the Ubiquinone and other terpenoid-quinone biosynthesis pathway exhibited positive correlations with IL-4 and T-AOC.

Within immune-related signaling pathways, the T cell receptor signaling pathway was negatively correlated with serum DAO and TNF-α levels, but positively correlated with IgG, IL-2, and IL-10. The Phagosome pathway showed positive correlations with IL-4 and T-AOC. Meanwhile, the TGF-β signaling pathway was negatively correlated with MDA, TNF-α, and IgA, while positively correlated with IgG and IL-2.

Collectively, these results indicate that alterations in gut microbial functional pathways are closely associated with host antioxidant status and immune responses, suggesting a coordinated interaction between microbial metabolic functions and systemic physiological regulation in calves.

## 4. Discussion

Early-stage diarrhea in calves represents a major health challenge that compromises not only early growth and development but also subsequent productive performance. Beyond acute disturbances in fluid and electrolyte balance, diarrhea can exert prolonged adverse effects by persistently impairing gastrointestinal maturation, immune homeostasis, and metabolic function [25,26]. Accumulating evidence indicates that diarrhea during early life is associated with reduced feed intake, impaired nutrient absorption, elevated oxidative stress, and disruption of intestinal microbial ecology, thereby establishing a self-perpetuating cycle of “gut dysfunction–immune dysregulation–metabolic disorder” [27,28]. Unlike general oxidative stress conditions such as heat stress or transport stress, diarrhea-associated oxidative stress is primarily driven by intestinal barrier disruption and endotoxin (LPS) translocation, which activates mucosal immune signaling pathways and amplifies ROS production locally within the gut. Therefore, oxidative stress in diarrheic calves should be considered a gut-originated pathological cascade rather than a systemic nonspecific stress response [29,30]. Consequently, the development of nutritional strategies capable of simultaneously modulating gut health, immune function, and microbial homeostasis is of considerable importance for facilitating recovery and improving growth performance in diarrheic calves. These observations are consistent with previous studies in neonatal ruminants and monogastrics, although the relative contribution of microbial dysbiosis, oxidative stress, and immune activation varies depending on pathogen type, diet composition, and host developmental stage [31].

In the present study, the effects of compound yeast culture supplementation on growth performance, antioxidant capacity, immune function, fecal characteristics, and gut microbiota composition and function were systematically evaluated in diarrheic calves. The results demonstrated that compound yeast culture exerted broad regulatory effects, as evidenced by increased feed intake and ADG during the mid-to-late stages of the trial, attenuation of oxidative stress and inflammatory responses, sustained improvement in fecal consistency, and a progressive shift in gut microbial structure and function toward a healthier profile. Importantly, these beneficial outcomes were not attributable to a single regulatory pathway, but rather reflected coordinated, multi-level interactions involving intestinal development, immune modulation, antioxidant defense, and microbial ecosystem reconstruction, collectively contributing to the restoration of physiological homeostasis in diarrheic calves. These findings are consistent with previous reports on yeast-based feed additives in ruminants [13,32,33], but extend them by integrating host phenotype, oxidative stress, immune response, and gut microbiota functional profiling into a unified mechanistic framework.

In summary, this study provides integrated phenotypic, functional, and microbiological evidence elucidating the mechanisms by which compound yeast culture supplementation mitigates calf diarrhea and its secondary consequences. These findings support the rational application of compound yeast cultures as a nutritional strategy for the prevention and control of calf diarrhea and for promoting sustainable, healthy calf rearing.

### 4.1. Effects of Compound Yeast Culture on Growth Performance in Diarrhoeic Calves

Early-stage diarrhea in calves is a major health challenge that compromises growth, development, and subsequent productive performance [34]. In addition to inducing acute disturbances in fluid and electrolyte balance, diarrhea is frequently accompanied by reduced feed intake and impaired nutrient absorption, resulting in sustained growth retardation [35,36]. In the present study, diarrheic calves without nutritional intervention exhibited persistently lower feed intake and ADG during the mid-to-late experimental period, further confirming the long-lasting inhibitory effects of early-life diarrhea on growth performance.

With respect to feed intake, compound yeast culture supplementation markedly improved average daily starter intake in diarrheic calves during both the mid-to-late phase of the trial (days 31–60) and across the entire experimental period (days 1–60), compared with diarrheic calves that were not supplemented. These findings suggest that the beneficial effects of compound yeast culture on feeding behavior are most pronounced during the gradual recovery phase of gastrointestinal function. Reduced feed intake in diarrheic calves is not solely attributable to anorexia but is also mediated by inflammatory cytokines such as TNF-α and IL-1β, together with impaired rumen fermentation, which collectively suppress appetite regulation and energy-sensing pathways [37]. Previous studies have demonstrated that bioactive metabolites and functional components present in yeast culture can optimize the rumen microenvironment and promote the restoration of rumen fermentation activity, thereby enhancing appetite and feeding capacity [32,33]. This mechanism is consistent with the significant increase in feed intake observed during the later stages of the current study.

Changes in ADG further supported these observations. Diarrheic calves receiving compound yeast culture exhibited significantly higher ADG than the diarrheic control group during days 31–60 and cumulatively over the entire trial period. This indicates that compound yeast culture supplementation not only stimulates feed intake but also improves the efficiency with which ingested nutrients are converted into body mass [38,39]. Given that body weight represents a cumulative parameter with limited sensitivity to short-term nutritional interventions, the improvement in ADG provides a more sensitive and robust indicator of enhanced growth efficiency, despite the absence of significant differences in body weight at individual sampling points.

The observed improvements in growth performance can be further interpreted in light of previous findings from our research group. Earlier studies have shown that compound yeast culture promotes rumen epithelial cell proliferation and accelerates the development of rumen papillae, thereby enhancing the structural basis for nutrient absorption [14,40]. In addition, compound yeast culture has been shown to modulate rumen microbial communities by increasing the relative abundance of fiber-degrading and volatile fatty acid–producing genera, including *Prevotella*, *Fibrobacter*, *Butyrivibrio*, and *Ruminococcus*, resulting in enhanced rumen fermentation capacity and elevated concentrations of total volatile fatty acids, acetate, propionate, and butyrate. Concurrently, the expression of key genes involved in epithelial nutrient transport and absorption—such as *MCT1*, *NHE1*, *NHE3*, *PAT1*, and *vH^+^-ATPase*—was significantly upregulated [41]. Collectively, these changes contribute to improved absorption and utilization of fermentation-derived energy substrates, thereby supporting enhanced growth performance.

In conclusion, under conditions of early-life diarrhea, compound yeast culture supplementation promotes rumen development and fermentation efficiency, leading to improved nutrient absorption and utilization. These effects translate into increased feed intake and ADG during the mid-to-late stages of recovery, ultimately facilitating the restoration and enhancement of growth performance in diarrheic calves.

### 4.2. Effects of Compound Yeast Culture on Antioxidant Capacity, Immune Function, and Faecal Characteristics in Diarrhoeic Calves

Early-life diarrhea in calves represents more than a transient manifestation of gastrointestinal dysfunction. Its initiation and progression are commonly accompanied by a cascade of pathological alterations, including disruption of intestinal barrier integrity, excessive activation of inflammatory responses, and elevation of systemic oxidative stress [5,42]. Following diarrhea onset, pathogenic microorganisms and their metabolites compromise epithelial structure, stimulate the release of pro-inflammatory mediators, and promote excessive production of reactive oxygen species (ROS), ultimately forming a self-perpetuating cycle characterized by “inflammation–oxidative stress–barrier dysfunction” [43,44]. In the present study, diarrheic calves exhibited persistently elevated fecal scores, dysregulated antioxidant indices, and impaired immune function at multiple stages, confirming the widespread involvement of these pathological processes in calf diarrhea.

Importantly, the pathological role of oxidative stress in diarrhea is mechanistically distinct from other stress conditions. In diarrheic calves, intestinal epithelial disruption enables lipopolysaccharide (LPS) translocation, which activates TLR4/NF-κB signaling and induces excessive ROS production in immune and epithelial cells [45]. At the same time, diarrhea-induced malabsorption reduces the intake of antioxidant micronutrients such as selenium, zinc, and vitamins A and E, resulting in weakened endogenous antioxidant defense. This dual mechanism of enhanced ROS generation combined with reduced antioxidant capacity explains why oxidative stress is particularly pronounced and persistent in diarrheal disease compared with other physiological stressors.

Fecal score dynamics provided a direct and sensitive indicator of intestinal recovery. Compared with non-supplemented diarrheic calves, compound yeast culture supplementation significantly reduced fecal scores during the 7–15 d and 16–30 d periods. Notably, by days 16–30, fecal scores in the yeast-supplemented group had recovered to levels comparable to those of healthy calves. These findings suggest that yeast culture does not merely suppress diarrheal symptoms acutely but facilitates the gradual restoration of intestinal function by improving the intestinal microenvironment [46]. Potential mechanisms include modulation of ruminal and intestinal microbial communities, reduction in harmful metabolite accumulation, and optimization of digestive and absorptive conditions, thereby decreasing the risk of persistent diarrhea [13,47].

Oxidative stress represents a central pathological component throughout the course of diarrhea. In the early-to-mid stages of the trial, diarrheic calves displayed elevated activities of SOD and GSH-Px concomitant with reduced T-AOC and increased MDA levels. This pattern indicates a compensatory activation of antioxidant enzyme systems under diarrheal stress, yet insufficient overall antioxidant capacity to effectively counteract excessive lipid peroxidation [48]. Although increased antioxidant enzyme activity has been reported in some enteric disease models, other studies have shown inconsistent responses depending on pathogen type, infection severity, and disease stage, suggesting that antioxidant defense in diarrhea is dynamic, shifting from early compensatory activation to later exhaustion under sustained oxidative stress [5]. With continued yeast culture supplementation, calves in the DIA-YC group exhibited significantly increased T-AOC and markedly reduced MDA concentrations during the mid-to-late stages, returning to levels comparable to those observed in CON. These results indicate that yeast culture enhances systemic antioxidant defense and alleviates oxidative damage [49]. Mechanistically, this effect may be attributed to the synergistic actions of bioactive components in yeast culture, such as β-glucans, B vitamins, and small peptides, which enhance antioxidant enzyme activity, as well as indirect effects arising from improved gut health and reduced inflammatory and metabolic stress [50]. These findings are consistent with previous studies by our group in beef cattle, which demonstrated that yeast culture supplementation significantly increased serum T-AOC, T-SOD, and GSH-Px activities, thereby improving redox homeostasis [12].

Compared with bacterial probiotics such as Lactobacillus, Bifidobacterium, and Leuconostoc mesenteroides, which mainly act through pathogen inhibition and competitive exclusion, yeast culture exerts a broader regulatory effect by providing bioactive cell wall components, including β-glucans and mannan oligosaccharides, as well as B vitamins and fermentation-related metabolites [33]. Notably, Leuconostoc mesenteroides has been reported to alleviate calf diarrhea and improve gut microbial balance [51], but its effects are generally strain-dependent and primarily restricted to microbial antagonism [52]. In contrast, yeast culture appears to regulate both intestinal microbial ecology and host systemic antioxidant capacity simultaneously, suggesting a more integrated mode of action.

Alterations in immune-related parameters further elucidated the regulatory role of compound yeast culture during diarrhea recovery. Elevated TNF-α levels, together with increased DAO and ET concentrations in early-stage diarrheic calves, reflected excessive inflammatory activation and increased intestinal permeability [53]. Following compound yeast culture supplementation, the DIA-YC group exhibited increased IgG concentrations, restoration of IL-2 levels, reduced TNF-α, and a pronounced elevation in the anti-inflammatory cytokine IL-10 during the mid-to-late stages. These changes indicate a progressive shift in the immune response from a pro-inflammatory state towards immune regulation and tissue repair. Functionally, immunomodulatory components such as β-glucans in yeast culture can be recognized by immune cells, thereby enhancing both humoral and innate immune responses [54]. Importantly, oxidative stress and immune responses are tightly interconnected biological processes. Excessive ROS can activate inflammatory signaling pathways such as NF-κB and NLRP3 inflammasome, while inflammatory cytokines further amplify ROS production. Therefore, the simultaneous reduction in MDA and TNF-α observed in this study suggests that compound yeast culture regulates a shared upstream inflammatory–oxidative axis rather than independent pathways [55]. Previous studies from our group have shown that yeast culture supplementation significantly increased serum IgG, IgA, IgM, lysozyme activity, and pro-inflammatory cytokines in beef cattle, reflecting enhanced immune responsiveness [12]. In dairy cattle, compound yeast culture further improved non-specific immunity by increasing lysozyme content, acid phosphatase activity, lymphocyte proliferative capacity, and neutrophil phagocytic activity [56]. These findings provide strong mechanistic support for the immune-modulatory effects observed in the present study.

Collectively, the beneficial effects of compound yeast culture in diarrheic calves are multi-dimensional rather than attributable to a single physiological pathway. By simultaneously enhancing antioxidant defense, modulating immune responses, and promoting repair of the intestinal barrier, yeast culture effectively alleviates diarrhea-induced physiological damage [57]. Enhanced antioxidant capacity mitigates oxidative injury to intestinal epithelial and immune cells, while suppression of excessive inflammation and reinforcement of immune regulatory functions facilitate the restoration of barrier integrity, ultimately manifesting as improved fecal consistency and reduced diarrhea severity.

In summary, the present findings are highly consistent with previous observations in beef and dairy cattle, demonstrating that compound yeast culture supplementation improves intestinal health through coordinated regulation of antioxidant capacity and immune function. Together, these results provide robust mechanistic and theoretical support for the application of compound yeast cultures in the prevention and control of calf diarrhea and in promoting sustainable, health-oriented calf rearing systems.

### 4.3. Effects of Compound Yeast Culture on Gut Microbiota Composition and KEGG Functions in Diarrhoeic Calves

Early-stage calf diarrhea is widely recognized as a multifactorial disorder arising from gut microbial dysbiosis, impaired intestinal barrier integrity, immune dysfunction, and excessive oxidative stress. Accumulating evidence indicates that diarrhea markedly reduces gut microbial diversity, weakens colonization resistance of beneficial microbes, and facilitates the expansion of opportunistic pathogens, thereby aggravating intestinal inflammation and systemic metabolic disturbances [58,59]. In the present study, we systematically characterized the structural and functional alterations of the gut microbiota in diarrheic calves and elucidated their associations with host antioxidant and immune phenotypes, providing novel mechanistic insights into the microbiota-mediated protective effects of compound yeast culture supplementation. Similar microbial shifts have been reported following probiotic supplementation; however, bacterial probiotics mainly exert narrow-spectrum colonization resistance, whereas yeast culture induces broader ecological restructuring by enhancing substrate availability for multiple microbial functional guilds [60,61].

At the community level, diarrhea significantly reduced microbial richness, as reflected by a lower Chao index during the early stage of the trial, and further induced pronounced disruptions in microbial diversity and community structure by day 30. β-diversity analyses demonstrated a clear separation between the gut microbial communities of diarrheic calves and CON, indicating that diarrhea induces global restructuring of the gut microbiome rather than isolated changes in individual taxa [62]. Notably, compound yeast culture supplementation markedly shifted the gut microbial community of diarrheic calves towards that of CON in PCA space, suggesting a substantial restorative effect on overall microbial ecosystem stability.

At the taxonomic level, diarrheic calves exhibited significant depletion of multiple genera associated with intestinal health and metabolic homeostasis, including *Ruminococcus* and *Parabacteroides*, accompanied by an increased relative abundance of potentially detrimental taxa such as *B. pullicaecorum* and *C. nexile*. These alterations may impair complex carbohydrate utilization and short-chain fatty acid production, thereby compromising intestinal barrier function and immune regulation [63,64]. In contrast, compound yeast culture supplementation significantly enriched several functionally beneficial genera, including *Phocaeicola*, *Prevotella*, *Coprococcus*, *Ruminococcus*, and *Parabacteroides*. These taxa are closely linked to energy metabolism, anti-inflammatory activity, and immune modulation [13,65], indicating that compound yeast culture promotes a shift towards a more functionally favorable gut microbial configuration. For instance, Leuconostoc mesenteroides has been shown to reduce diarrhea severity and modulate gut microbiota composition in calves [51], but its effects are largely restricted to pathogen suppression, whereas yeast culture additionally regulates host metabolic and antioxidant systems via microbiota-derived metabolites [60].

Consistent with these compositional changes, KEGG functional enrichment analysis revealed that diarrhea profoundly altered gut microbial functional profiles. Pathways related to pathogenic infection, bacterial motility, and ET biosynthesis were significantly enriched, whereas multiple pathways associated with antioxidant defense and metabolic homeostasis were suppressed. These functional disruptions closely paralleled the elevated oxidative stress and impaired immune status observed in diarrheic calves, supporting the notion that microbial functional dysregulation contributes to systemic inflammation and redox imbalance [3,66,67]. Importantly, compound yeast culture supplementation markedly enhanced several immune- and antioxidant-related functional pathways, including the T cell receptor signaling pathway, phagosomes, TGF-β signaling pathway, oxidative phosphorylation, riboflavin metabolism, and ubiquinone, as well as other terpenoid-quinone biosynthesis pathways. These pathways are critically involved in immune cell activation, anti-inflammatory regulation, mitochondrial energy metabolism, and reactive oxygen species scavenging [68,69,70,71]. These findings indicate that compound yeast culture not only reshapes gut microbial composition but also functionally reprograms the microbiome to better support host immune and antioxidant demands.

Further Spearman correlation analyses provided direct evidence for a coordinated ‘microbiota–function–host phenotype’ relationship. Several genera enriched in the compound yeast culture-supplemented group—such as *Ruminococcus*, *Parabacteroides*, *Prevotella*, and *Coprococcus*—exhibited significant positive correlations with serum antioxidant and immune markers, including T-AOC, SOD, GSH-Px, IgG, and IL-2, while showing negative correlations with oxidative stress and pro-inflammatory indicators such as MDA and TNF-α. In parallel, multiple immune- and antioxidant-related KEGG pathways displayed highly consistent correlation patterns with these serum parameters, reinforcing the functional relevance of microbiota-driven host regulation [72]. Taken together, these findings support a multi-layer regulatory model in which compound yeast culture alleviates diarrhea through restoration of microbial ecological balance, enhancement of microbial metabolic outputs such as SCFAs, and modulation of host immune and antioxidant signaling pathways. This “microbiota–metabolite–host axis” provides a more comprehensive explanation than the traditional single-pathway probiotic hypothesis.

In summary, diarrhea induces concurrent structural and functional dysbiosis of the calf gut microbiota, thereby exacerbating oxidative stress and immune imbalance. Compound yeast culture supplementation promotes microbiome reconstitution by reshaping microbial community structure and enhancing immunoregulatory and antioxidant-related functional pathways, ultimately facilitating the restoration of host immune homeostasis and metabolic equilibrium. This synergistic ‘microbiota–function–host phenotype’ regulatory mechanism provides a robust theoretical basis for the application of compound yeast culture in the prevention and control of calf diarrhea.

## 5. Conclusions

This study demonstrates that CYC supplementation effectively promotes recovery from early-life diarrhea in calves, as evidenced by improved feed intake, growth performance, antioxidant capacity, and immune function. These benefits are closely associated with the restoration of gut microbiota composition and functional potential.

Importantly, CYC alleviates diarrhea and facilitates health recovery through coordinated modulation of the gut microbiota–functional pathways–host phenotype axis, enhancing microbial functions related to immunity and antioxidant activity and their interactions with host physiological responses.

Overall, these findings provide mechanistic insights and scientific support for the application of CYC in improving calf health and promoting sustainable ruminant production.

## Figures and Tables

**Table 1:** Feeding amount of regular milk for calves (L/time).

Age (Days)	Feeding Amount
7~10	2.0
11~20	4.0
21~25	5.0
26~57	6.0
58~62	4.5
63	4.0
64	3.5
65	3.0
66	2.5
67	2.0

**Table 2:** Composition and nutrient levels of starter (%, DM basis).

Ingredients	Content
Corn grain	57.7
Soybean meal	19.3
Cottonseed meal	15.0
Wheat bran	3.0
Premix ^1^	5.0
Total	100.0
Nutrient levels ^2^	
NE_mf_/(MJ/kg)	5.61
DM	91.6
CP	20.03
ADF	14.07
NDF	24.47
Ca	0.81
P	0.51

^1^ The premix provided the following per kg of pellet dies: VA 250,000 IU, VD 60,000 IU, VE 1000 lU, Fe 80 mg, Cu 12 mg, Zn 80 mg, Mn 70 mg, Se 0.40 mg, I 1 mg, Co 0.80 mg. ^2^ NE_mf_ was calculated by reference to NASEM (2016) [18], while the others were measured values.

**Table 3:** Scoring standard of calf feces.

Appearance	Score Standard	Score
Normal	Well-formed and not loose	1
Formed	Soft but formable	2
Unformed	Loose, paste-like	3
Unformed	Watery, easy to spread	4
Unformed	Watery, separated fecal matter, splashing	5

**Table 4:** Serum antioxidant indicator test kits and their product numbers and measurement methods.

Item	Kit Name	Method	Product Number
T-AOC	Total antioxidant capacity assay kit	FRAP	RXFG0297
SOD	Superoxide Dismutase (SOD) assay kit	NBT	RXWB0482
GSH-Px	Glutathione Peroxidase (GSH-Px) assay kit	Visible Color Development Method	RXWB0100
MDA	Malondialdehyde (MDA) assay kit	Micromethod	RXWB0005

**Table 5:** Serum immunological indicator test kits and their product numbers and testing methods.

Item	Kit Name	Method	Product Number
IgG	Immunoglobulin G Assay Kit	ELISA	RX1600039B
IgA	Immunoglobulin A Assay Kit	ELISA	RX1600805B
IL-2	Interleukin-2 Assay Kit	ELISA	RX1600855B
IL-10	Interleukin-10 Assay Kit	ELISA	RX1600646B
IL-4	Interleukin-4 Assay Kit	ELISA	RX1600854B
TNF-α	Bovine Tumor Necrosis Factor-Alpha (TNF-α) Kit	ELISA	RX1600738B
DAO	Bovine Diamine Oxidase (DAO) ELISA Kit	ELISA	RX2D779536
ET	Bovine Endothelin (ET) Quantitative ELISA Kit	ELISA	JRXW774866

**Table 6:** Effects of CYC on growth performance of calves.

Item	Time Point	CON	DIA	DIA-YC	SEM	*p*-Value
ADFI (g/d)	Day 1~30	75.11	69.69	82.95	3.77	0.375
	Day 31~60	329.77 ^ab^	315.14 ^a^	360.67 ^b^	6.68	0.008
	Day 1~60	202.44 ^a^	192.41 ^a^	221.81 ^b^	4.00	0.003
Body weight/kg	Day 1	38.50	38.33	38.17	0.67	0.982
	Day 30	59.67	59.13	59.57	0.86	0.969
	Day 60	86.86	84.94	88.08	1.00	0.657
ADG (Kg/d)	Day 1~30	0.71	0.69	0.71	0.03	0.974
	Day 31~60	0.91 ^ab^	0.86 ^a^	0.95 ^b^	0.01	0.013
	Day 1~60	0.81 ^ab^	0.78 ^a^	0.83 ^b^	0.01	0.011

In the same row, values with no letter or the same letter superscripts show no significant difference (*p* > 0.05), while values with different small letter superscripts show a significant difference (*p* < 0.05). The value is the mean ± SEM, *n* = 12. ADG = average daily gain. ADFI = average daily feed intake.

**Table 7:** Effects of CYC on fecal score in calves.

Item	Time Point	CON	DIA	DIA-YC	SEM	*p*-Value
Fecal score/score	Day 1~7	1.43 ^a^	3.21 ^b^	2.93 ^b^	0.20	<0.01
	Day 7~15	1.50 ^a^	2.88 ^b^	2.27 ^c^	0.15	<0.01
	Day 16~30	1.41 ^a^	2.33 ^b^	1.81 ^a^	0.34	0.021
	Day 31~60	1.48	1.85	1.59	0.55	0.216

In the same row, values with no letter or the same letter superscripts show no significant difference (*p* > 0.05), while values with different small letter superscripts show a significant difference (*p* < 0.05). The value is the mean ± SEM, *n* = 12.

**Table 8:** Effects of CYC on antioxidant capacity in calves.

Item	Time Point	CON	DIA	DIA-YC	SEM	*p*-Value
T-AOC (µg/mL)	Day 1	6.39	5.86	5.83	0.106	0.033
	Day 7	6.45 ^a^	5.79 ^b^	6.17 ^ab^	0.109	0.025
	Day 15	6.47 ^a^	5.94 ^b^	6.43 ^a^	0.09	0.008
	Day 30	6.24 ^a^	6.04 ^a^	7.46 ^b^	0.23	0.006
SOD (U/mL)	Day 1	44.59	50.5	56.9	2.3	0.079
	Day 7	49.55 ^a^	85.49 ^b^	81.01 ^b^	5.21	<0.001
	Day 15	48.3 ^a^	88.01 ^b^	99.04 ^b^	7.02	<0.001
	Day 30	43.05 ^a^	70.0 ^b^	82.83 ^b^	5.41	<0.001
GSH-Px (µmol/mL)	Day 1	500.15 ^a^	545.09 ^b^	546.15 ^b^	8.15	0.012
	Day 7	517.75 ^a^	563.93 ^ac^	574.69 ^bc^	9.51	0.014
	Day 15	521.76 ^a^	591.02 ^b^	584.54 ^b^	10.65	0.001
	Day 30	531.04 ^a^	595.66 ^b^	603.63 ^b^	11.1	0.001
MDA (nmol/mL)	Day 1	12.12	14.82	14.12	0.55	0.101
	Day 7	8.48 ^a^	15.94 ^b^	14.23 ^b^	1.06	<0.001
	Day 15	5.73 ^a^	9.47 ^b^	7.99 ^ab^	0.55	0.004
	Day 30	5.47 ^a^	7.71 ^b^	5.62 ^a^	0.39	0.012

In the same row, values with no letter or the same letter superscripts show no significant difference (*p* > 0.05), while values with different small letter superscripts show a significant difference (*p* < 0.05). The value is the mean ± SEM, *n* = 6.

**Table 9:** Effects of CYC on immune function in calves.

Item	Time Point	CON	DIA	DIA-YC	SEM	*p*-Value
IgG (mg/mL)	Day 1	1.69 ^a^	1.41 ^b^	1.42 ^b^	0.048	0.009
	Day 7	1.88	1.79	1.85	0.032	0.628
	Day 15	1.98	1.89	2.09	0.038	0.113
	Day 30	2.31 ^ab^	2.11 ^a^	2.36 ^b^	0.046	0.038
IgA (μg/mL)	Day 1	461.06	438.46	439.87	8.65	0.535
	Day 7	547.13	538.91	563.75	5.78	0.214
	Day 15	664.06	674.59	622.15	12.56	0.207
	Day 30	608.78 ^ac^	731.74 ^b^	626.82 ^c^	19.59	0.005
IL-2 (pg/mL)	Day 1	128.14	116.29	117.58	3.80	0.416
	Day 7	150.17	131.74	140.57	3.77	0.132
	Day 15	162.23 ^ab^	150.68 ^a^	174.69 ^b^	3.76	0.013
	Day 30	236.51 ^a^	196.94 ^b^	243.77 ^a^	7.23	0.003
TNF-α (pg/mL)	Day 1	41.15 ^a^	50.43 ^b^	52.20 ^b^	1.65	0.001
	Day 7	37.46 ^a^	45.94 ^b^	45.14 ^b^	1.51	0.019
	Day 15	34.53	38.00	35.43	0.85	0.239
	Day 30	29.60 ^a^	37.16 ^b^	30.43 ^a^	1.29	0.012
IL-10 (pg/mL)	Day 1	23.09	26.89	26.06	0.78	0.107
	Day 7	22.28 ^a^	30.73 ^b^	33.63 ^b^	1.73	0.004
	Day 15	26.57 ^a^	34.46 ^b^	37.84 ^b^	1.61	0.001
	Day 30	26.89 ^a^	28.21 ^a^	40.97 ^b^	2.20	0.002
IL-4 (pg/mL)	Day 1	7.19	7.13	7.11	0.16	0.982
	Day 7	7.68	7.82	8.11	0.15	0.552
	Day 15	8.41	8.75	9.18	0.21	0.332
	Day 30	9.28	9.11	9.98	0.19	0.168
DAO (ng/mL)	Day 1	42.56 ^a^	50.39 ^b^	52.65 ^b^	1.61	0.009
	Day 7	45.31 ^a^	51.56 ^b^	46.20 ^ab^	1.13	0.029
	Day 15	44.43	46.01	43.48	1.02	0.64
	Day 30	43.74	43.29	40.05	0.87	0.172
ET (pg/mL)	Day 1	15.71 ^a^	32.31 ^b^	33.23 ^b^	2.55	<0.001
	Day 7	16.09 ^a^	24.12 ^b^	20.21 ^ab^	1.25	0.012
	Day 15	18.79	17.15	16.30	0.61	0.260
	Day 30	15.17	15.16	14.64	0.59	0.930

In the same row, values with no letter or the same letter superscripts show no significant difference (*p* > 0.05), while values with different small letter superscripts show a significant difference (*p* < 0.05). The value is the mean ± SEM, *n* = 6.

## Data Availability

The original contributions presented in this study are included in the article/Supplementary Material. Further inquiries can be directed to the corresponding author.

## Supplementary Materials

The following supporting information can be downloaded at: https://www.mdpi.com/article/10.3390/microorganisms14050995/s1; File S1: KEGG Pathway Level 3 (Pathway ID) enrichment analysis of CON30 vs. DIA30 groups. File S2: KEGG Pathway Level 3 (Pathway ID) enrichment analysis of DIA30 vs DIA_YC30 groups.

## Abbreviations

The following abbreviations are used in this manuscript:

ADFI	average daily feed intake
ADG	average daily gain
ANOVA	analysis of variance
ASV	amplicon sequence variant
CAT	catalase
CON	healthy control group
CYC	compound yeast culture
DIA	diarrheic calves without supplementation
DIA-YC	diarrheic calves supplemented with compound yeast culture
GSH-Px	glutathione peroxidase
IgA	immunoglobulin A
IgG	immunoglobulin G
IgM	immunoglobulin M
IL	interleukin
KEGG	Kyoto Encyclopedia of Genes and Genomes
LPS	lipopolysaccharide
PCoA	principal coordinates analysis
ROS	reactive oxygen species
SEM	standard error of the mean
SOD	superoxide dismutase
TNF-α	tumor necrosis factor alpha
YC	yeast culture

## Appendix A

**Table A1:** Fermentation substrate composition.

Item	Content
Wheat bran (%)	15
Spraying corn bran (%)	12
Corn (%)	16
Rice bran (%)	10
DDGS (%)	8
Corn germ meal (%)	27
Soybean meal (%)	12

**Table A2:** Nutritional components of compound yeast culture.

Item	Yeast Culture
Dry matter (%)	≥88
Crude protein (%)	≥20
Crude ash (%)	≤9
Neutral detergent fiber (%)	≤34
Acid detergent fiber (%)	≤20
Live yeast cells (cfu/g)	≥10^6^
β-glucan (%)	≥0.5
mannan (%)	≥0.5
